# Palmitate and insulin synergistically induce IL-6 expression in human monocytes

**DOI:** 10.1186/1475-2840-9-73

**Published:** 2010-11-05

**Authors:** Robert C Bunn, Gael E Cockrell, Yang Ou, Kathryn M Thrailkill, Charles K Lumpkin, John L Fowlkes

**Affiliations:** 1 Department of Pediatrics, University of Arkansas for Medical Sciences, Little Rock, Arkansas, USA

## Abstract

**Background:**

Insulin resistance is associated with a proinflammatory state that promotes the development of complications such as type 2 diabetes mellitus (T2DM) and atherosclerosis. The metabolic stimuli that initiate and propagate proinflammatory cytokine production and the cellular origin of proinflammatory cytokines in insulin resistance have not been fully elucidated. Circulating proinflammatory monocytes show signs of enhanced inflammation in obese, insulin resistant subjects and are thus a potential source of proinflammatory cytokine production. The specific, circulating metabolic factors that might stimulate monocyte inflammation in insulin resistant subjects are poorly characterized. We have examined whether saturated nonesterified fatty acids (NEFA) and insulin, which increase in concentration with developing insulin resistance, can trigger the production of interleukin (IL)-6 and tumor necrosis factor (TNF)-α in human monocytes.

**Methods:**

Messenger RNA and protein levels of the proinflammatory cytokines IL-6 and TNF-α were measured by quantitative real-time PCR (qRT-PCR) and Luminex bioassays. Student's *t*-test was used with a significance level of *p *< 0.05 to determine significance between treatment groups.

**Results:**

Esterification of palmitate with coenzyme A (CoA) was necessary, while β-oxidation and ceramide biosynthesis were not required, for the induction of IL-6 and TNF-α in THP-1 monocytes. Monocytes incubated with insulin and palmitate together produced more IL-6 mRNA and protein, and more TNF-α protein, compared to monocytes incubated with palmitate alone. Incubation of monocytes with insulin alone did not affect the production of IL-6 or TNF-α. Both PI3K-Akt and MEK/ERK signalling pathways are important for cytokine induction by palmitate. MEK/ERK signalling is necessary for synergistic induction of IL-6 by palmitate and insulin.

**Conclusions:**

High levels of saturated NEFA, such as palmitate, when combined with hyperinsulinemia, may activate human monocytes to produce proinflammatory cytokines and support the development and propagation of the subacute, chronic inflammatory state that is characteristic of insulin resistance. Results with inhibitors of β-oxidation and ceramide biosynthesis pathways suggest that increased fatty acid flux through the glycerolipid biosynthesis pathway may be involved in promoting proinflammatory cytokine production in monocytes.

## Background

Insulin resistance is characterized by a myriad of metabolic abnormalities, including hyperinsulinemia, hypertriglyceridemia, and an increased concentration of NEFA in blood [1]. These dysmetabolic features, sometimes referred to as the metabolic syndrome, are believed to contribute to the development of severe complications of insulin resistance, such as T2DM and atherosclerotic heart disease [2]. A common feature observed in subjects with insulin resistance, T2DM, and atherosclerotic heart disease is chronic, low-grade, systemic inflammation [3,4] as evidenced by increases in the concentration of proinflammatory cytokines (e.g., IL-6) in the blood, as well as increased concentrations in the blood of surrogate markers for systemic IL-6 bioactivity, such as C-reactive protein. The metabolic stimuli responsible for the increase in circulating proinflammatory cytokines and the cellular source of these cytokines in insulin resistant subjects are not well understood.

Adipose tissue has garnered a great deal of attention as a potential source of elevated circulating inflammatory cytokines in obesity and insulin resistance due to many studies demonstrating that adipose tissue can synthesize and secrete pro-inflammatory cytokines, including TNF-α [5,6] and IL-6 [7]. Recently it was shown that increased numbers of macrophages accumulate in adipose tissue in the obese [8], and these macrophages likely account for much of the inflammatory cytokine secretion from adipose tissue. However, it was reported that subcutaneous adipose tissue does not release TNF-α *in vivo*, and likely accounts for only 15-35% of systemic IL-6 release [7]. Also, Kern *et al *[9] reported that IL-6 concentration in plasma was positively correlated with obesity and plasma NEFA levels, but adipose tissue IL-6 production was not strongly affected by obesity. Therefore, it is possible that the bulk of the systemic proinflammatory cytokines in the obese, insulin resistant state are derived from non-adipose cellular and tissue sources.

Adipose tissue macrophages and macrophages of atherosclerotic plaques presumably arise from circulating monocytes, a heterogeneous population of cells that in humans can be divided into three discrete subsets based on the expression level of cell surface markers CD14, CD16, and CD64 [10]. CD14^hi^CD16^- ^cells make up the majority of blood monocytes (~80%) and have a proinflammatory phenotype characterized by their ability to produce abundant quantities of cytokines such as TNF-α and IL-6 [11,12] when activated. An analogous subset of proinflammatory monocytes has been described in the mouse, albeit based on a distinct set of cell surface markers [13]. Cells of this monocyte subset in mice and humans also express high levels of receptors for chemotactic peptides (e.g., CCR2, the receptor for monocyte chemoattractant protein-1), allowing these cells to efficiently respond to localized sites of inflammation [12]. Indeed, it is the proinflammatory monocyte subset that accumulates preferentially in obese adipose tissue [14] and atherosclerotic plaques [15].

An emerging concept is that monocyte subsets may be committed to a specific function before they localize to sites of infection or tissue damage [12]. Evidence for activation of circulating blood monocytes into a proinflammatory phenotype includes studies showing that circulating monocytes isolated from obese human subjects contained greater amounts of inflammatory cytokine messenger RNA relative to monocytes isolated from lean subjects [16], and induced hyperlipidemia in mice is associated with expansion of the proinflammatory monocyte subpopulation [15]. Additionally, lipid infusion in humans acutely activates NFκB, a proinflammatory transcription factor, and stimulates the production of macrophage migration inhibitory factor and reactive oxygen species in circulating mononuclear leukocytes [17]. Conversely, activation of peroxisome proliferator-activated receptor γ (PPAR γ) has been shown to prime an anti-inflammatory subset of monocytes into an enhanced anti-inflammatory monocyte phenotype [18].

NEFA have been demonstrated to induce inflammatory cytokine production in mature macrophages [19]. However, the influence of NEFA on the inflammatory phenotype of monocytes has not been explored. Furthermore, the combined impact of NEFA and hyperinsulinemia, which is particularly relevant to the insulin resistant metabolic state [1], has not been explored for its effect on monocyte inflammation. In this study, we hypothesized that NEFA could act on human monocytes to induce a proinflammatory phenotype as judged by increased inflammatory cytokine production. We provide evidence that long-chain saturated fatty acids can stimulate production and release of prototypical proinflammatory cytokines IL-6 and TNF- α in monocytes. Furthermore, we demonstrate that insulin synergizes with palmitate to induce higher levels of IL-6 in monocytes than that induced by palmitate alone.

## Methods

### Materials

THP-1 human monocytic leukemia cells were obtained from American Type Culture Collection (Manassas, VA). Fatty acids (palmitate (C16:0), stearate (C18:0), methylpalmitate, 2-bromopalmitate) and essentially fatty acid-free, low endotoxin bovine serum albumin (BSA, Sigma A8806) were obtained from Sigma-Aldrich (St. Louis, MO). Regular human insulin (Humulin R100, Eli Lilly, Indianapolis, IN) was obtained from the research pharmacy at Arkansas Children's Hospital. Inhibitors were obtained from EMD Biosciences (San Diego, CA) or Sigma-Aldrich.

### Preparation of NEFA solutions

NEFA (50 mM) were dissolved in 0.1 M sodium hydroxide with brief heating at 70°C. NEFA-BSA complexes were prepared by slowly adding 0.2 ml of NEFA solutions to 0.8 ml of warm BSA (250 mg/ml in Dulbecco's phosphate buffered saline, pH 7.2) with stirring, to yield a 10 mM NEFA-BSA stock solution with an approximate 3:1 NEFA:BSA ratio. Solutions were incubated at 37°C for 10-15 minutes prior to use, adjusted to pH 7.2, and filter sterilized. Solutions were prepared fresh for each experiment. Endotoxin levels were determined in the 10 mM NEFA-BSA stock solution using a Pyrogent Plus Gel Clot assay according to the manufacturer's instructions (Lonza Walkersville, Walkersville, MD) and were less than 10 EU/ml.

### Cell Culture Conditions and Experiments

THP-1 cells were cultured in RPMI 1640 (Invitrogen, Carlsbad, CA) supplemented with 10% fetal bovine serum (Hyclone, Logan, UT), penicillin (100 U/ml)-streptomycin (100 μg/ml) (Sigma-Aldrich), 55 μM 2-mercaptoethanol (Invitrogen), and 10 mM Hepes, pH 7.55 (Invitrogen). Cells were maintained at a density between 1 - 8 × 10^5 ^cells/ml and used between passages 6 - 20.

Cells were seeded in serum free, supplemented RPMI 1640 containing 0.2% fatty acid free BSA at 5 × 10^5 ^cells per ml/well in 24 well plates. Cells were rendered quiescent by overnight incubation in serum free media before stimulation with NEFA. Media (1 mL) and cells were collected at various times following stimulation with NEFA. Wells were washed with 0.5 mL of Dulbecco's phosphate buffered saline and the wash was combined with the cells/media. Cells were pelleted at 500 × g for 5 minutes at 4°C. Cytokine protein concentrations were determined in media from cells stimulated for 12 or 24 hours with NEFA.

Human primary monocytes were isolated by adhesion from the peripheral blood mononuclear cell (PBMC) fraction of whole blood. Approximately 50 ml of heparinized whole blood was obtained from healthy human volunteers according to a research protocol approved by the University of Arkansas Institutional Review Board. Blood was layered over a Histopaque 1077 gradient (Sigma-Aldrich) and centrifuged for 30 minutes at 400 g. PBMC were removed from the Histopaque-plasma interface and were washed several times with a buffered saline solution. PBMC (2 × 10^6^) were plated in RPMI 1640 supplemented with 10% donor serum overnight. The next morning, non-adherent cells were washed from the plate and RPMI 1640 supplemented with 10% FBS was added. Treatments with NEFA and/or insulin were initiated approximately 8 hours later and were allowed to proceed for 24 hours.

### Western Blot Analysis of phosphorylated and total ERK1/2 and Akt

THP-1 cells were treated with insulin for 30 minutes, harvested by centrifugation for 10 seconds in a microcentrifuge and washed one time with ice-cold phosphate buffered saline. The cell pellet was lysed in SDS sample buffer (62.5 mM Tris-HCl (pH 6.8), 2% w/v sodium lauryl sulphate, 10% glycerol, 50 mM dithiothreitol, 0.01% bromophenol blue) and heated. Extracts were resolved on 10% SDS-polyacrylamide gels and transferred to polyvinylidene difluoride membranes. Membranes were incubated with polyclonal antibodies (Cell Signaling Technology, Danvers, MA) directed against human Akt, phosphorylated human Akt (phospho-threonine 308), human ERK1/2, and phosphorylated human ERK1/2 (ERK1 phospho-threonine 202, tyrosine 204, ERK2 phospho-threonine 185, phospho-tyrosine 187) and signals were developed according to the manufacturer's protocol. Images were captured on a VersaDoc 5000 (Bio-Rad).

### RNA Isolation and Real-Time Polymerase Chain Reaction

Cells were lysed in Tri Reagent (Sigma-Aldrich) and total RNA was prepared according to the manufacturer's protocol. RNA (5 μg) was treated with DNase I (Promega, Madison, WI) according to the manufacturer's protocol. DNase reactions were diluted 10-fold with nuclease free water (Invitrogen) and were concentrated in YM-100 spin columns (Millipore). RNA (0.5 μg) was reverse-transcribed using iScript (Bio-Rad, Hercules, CA) according to the manufacturer's protocol. Quantitative PCR was carried out in an Applied Biosystems Prism^® ^7900HT Sequence Detection System (California) using SYBR^® ^Green Supermix with ROX (Bio-Rad). Reactions contained 25 ng of cDNA and 0.25 μM forward and reverse primers. Primers were designed using Beacon Designer 3.0 (Premier Biosoft Intl., Palo Alto, CA) and were ordered from Sigma-Aldrich at standard desalted purity. Pooled THP-1 cDNA, from cells that were treated with 1 μg/ml bacterial lipopolysaccharide to induce cytokine production, was serially diluted from 25 ng to 8 pg to construct a standard curve for all genes of interest and for reference genes. Gene expression was normalized for GAPDH expression to accurately reflect input cDNA quantity. Primer sequences were GAPDH forward: 5' ggagtccactggcgtctt 3', GAPDH reverse: 5' aggctgttgtcatacttctcat 3', IL-6 forward: 5' ttcggtccagttgccttctc 3', IL-6 reverse: 5' gaggtgagtggctgtctgtg 3', TNF-α forward: 5' ctccaggcggtgccttgttc 3', TNF-α reverse: 5' caggcagaagagcgtggtg 3'.

### Determination of Secreted IL-6 and TNF-α protein

IL-6 and TNF-α were measured simultaneously in conditioned media prepared from THP-1 monocytes using a high sensitivity human cytokine multiplex immunoassay (Linco, St. Charles, MO). Assays were run on a Luminex^® ^100™ Bioanalyzer (Luminex Corp., Austin, TX) according to the kit manufacturer's instructions. Kits contained distinct groups of microspheres (each group bearing unique fluorescence intensity and a specific cytokine antibody), biotinylated cytokine antibodies, and phycoerythrin conjugated streptavidin. Conditioned media samples were incubated with antibody-coated microspheres, which bind to specific cytokines present in the media. Next, microsphere-cytokine complexes were washed and incubated with biotinylated cytokine antibodies, which bind to cytokines present on the microspheres. A final incubation was performed in which phycoerythrin-labeled streptavidin was allowed to bind to biotinylated IL-6 or TNF-α antibodies present on microspheres. Microspheres were then loaded into a Luminex^® ^100™ Bioanalyzer, which quantifies the amount of phycoerythrin fluorescence present on each of the distinct microsphere groups. At least 50 individual microspheres were counted for each cytokine, and the median fluorescence intensity was used for subsequent calculations.

### Statistical Analysis

Data are represented as the mean ± standard error (SE) for three to four independent measurements. Student's two-tailed unpaired *t-*test was used for comparisons between groups with significance set at *P *< 0.05.

## Results

### Saturated long-chain NEFA stimulate IL-6 and TNF-α production in THP-1 cells

THP-1 monocytic cells have been used extensively as a model of primary human monocytes, have been shown to closely mimic primary human monocytes in their production of TNF-α in response to lipopolysaccharide [20] and have been utilized in diabetes-related studies, such as studies to investigate the production of IL-6 by monocytes in hyperglycemic conditions [21]. Therefore, we chose to utilize the THP-1 cell line to study the possible role of NEFA in the induction of inflammation in human monocytes.

**Figure 1:**
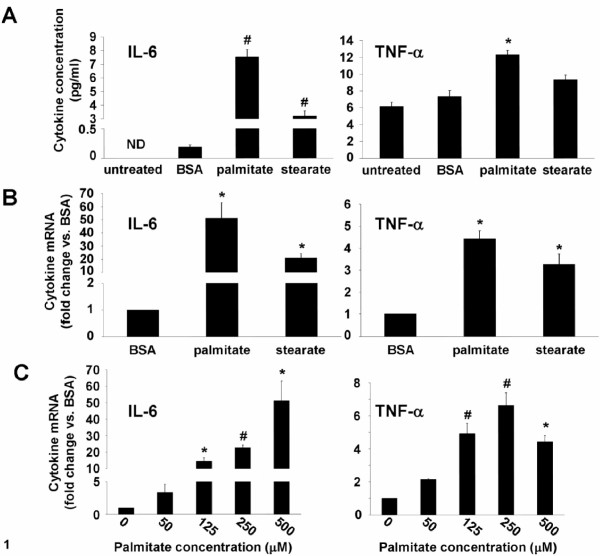
**Saturated NEFA stimulate IL-6 and TNF-α production**. *A*, IL-6 and TNF-α concentration in 24-hour conditioned media from THP-1 cells that were untreated or stimulated with 500 μM palmitate or stearate, or NEFA- and endotoxin-free BSA. *B*, IL-6 and TNF-α mRNA expression in THP-1 cells stimulated as in *A *for 12 hours. *C*, IL-6 and TNF-α mRNA expression in THP-1 cells stimulated with the indicated concentrations of palmitate for 12 hours. Values are means ± SE (n = 3-4). *, *p *< 0.05, # *p *< 0.01 *versus *BSA treatment, ND - below the limit of detection.

Normal circulating NEFA concentrations are less than 700 μM in the post-absorptive state [22], therefore, we chose to use 500 μM as an initial concentration of NEFA to determine the effect of long-chain saturated NEFA on inflammatory cytokine production in THP-1 monocytes. THP-1 cells that had been incubated for 24 hours with 500 μM palmitate, a 16 carbon saturated fatty acid, secreted significantly more IL-6 and TNF-α into the media compared to media from control cells incubated with BSA lacking bound NEFA (Figure 1A). Stearate, an 18 carbon saturated fatty acid, also stimulated IL-6 release, but did not increase the amount of TNF-α released over that released from untreated or BSA-treated control cells (Figure 1A).

Consistent with the increase in IL-6 protein production elicited by palmitate and stearate, IL-6 mRNA expression was increased significantly in THP-1 cells incubated for 12 hours with 500 μM palmitate (~50-fold induction) or stearate (~20-fold induction) compared to cells incubated with BSA alone (Figure 1B). While palmitate, but not stearate, stimulated TNF-α protein synthesis and release by THP-1 cells, both NEFA significantly increased TNF-α mRNA expression compared to cells treated with BSA alone (Figure 1B).

The concentration of NEFA in plasma can vary over a wide range depending on feeding status and sensitivity to the anti-lipolytic effects of insulin. To understand how IL-6 and TNF-α could be regulated by a range of physiological concentrations of NEFA in monocytes, THP-1 cells were treated with palmitate concentrations ranging from 50 μM to 500 μM. IL-6 and TNF- α mRNA expression was significantly induced by concentrations of palmitate ≥ 125 μM (Figure 1C), indicating that inflammatory cytokine production in monocytes may be significantly increased by conditions such as insulin resistance, where monocytes would be exposed to chronically elevated plasma NEFA concentrations.

### Palmitate metabolism is required for induction of IL-6 and TNF-α in THP-1 cells

**Figure 2:**
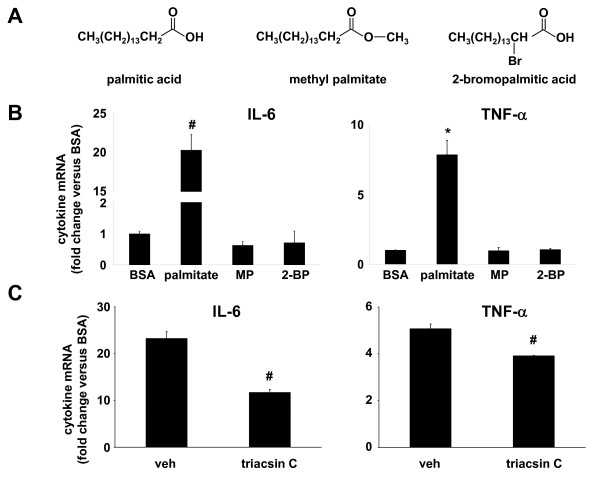
**Palmitate metabolism is required for the induction of IL-6 and TNF-α**. *A*, Chemical structure of palmitic acid and non-metabolizable palmitic acid analogs methylpalmitate (MP) and 2-bromopalmitate (2-BP). *B*, IL-6 and TNF-α mRNA expression in THP-1 cells stimulated for 12 hours with palmitate (250 μM) or equivalent concentrations of MP or 2-BP. *C*, IL-6 and TNF-α mRNA expression in THP-1 cells stimulated with palmitate in the presence of vehicle (DMSO) or an inhibitor of long chain fatty acyl CoA synthetase (triacsin C, 1 μM). Values are means ± SE (n = 3). *, *p *< 0.05, #, *p *< 0.01 *versus *BSA.

Two non-metabolizable analogs of palmitic acid, methyl palmitate and 2-bromopalmitic acid (Figure 2A), were utilized to determine whether intracellular metabolism of palmitate was required for the induction of IL-6 and TNF-α in monocytes. The esterification of palmitate by a methyl group in methyl palmitate prevents activation of this molecule by CoA, and abrogates downstream metabolism of the fatty acid, whereas 2-bromopalmitate can be activated by CoA, but cannot be further metabolized by β-oxidation or esterification with glycerol to form glycerolipids [23]. However, 2-bromopalmitate has been shown to occupy the same binding sites within albumin and fatty acid binding proteins, with a similar binding affinity, as unmodified palmitate [24]. Incubation with 250 μM palmitate for 12 hours stimulated monocytes to produce IL-6 and TNF-α mRNAs at levels >20-fold and >7-fold greater, respectively, than cells stimulated with BSA lacking bound NEFA (Figure 2B). In contrast, cells treated with either of the non-metabolizable palmitate analogs, at the same concentration used for palmitate, produced amounts of IL-6 and TNF-α mRNA that were not significantly increased compared to cells treated with BSA (Figure 2B), suggesting that palmitate metabolism via the glycerolipid biosynthetic pathway, ceramide biosynthetic pathway, or β-oxidation pathway is necessary for the induction of IL-6 and TNF- α in monocytes. Consistent with these results, we observed that triacsin C, a competitive inhibitor of fatty acid binding to long-chain fatty acyl CoA synthetases, significantly inhibited the induction of IL-6 and TNF-α mRNAs in cells incubated with palmitate (Figure 2C).

**Figure 3:**
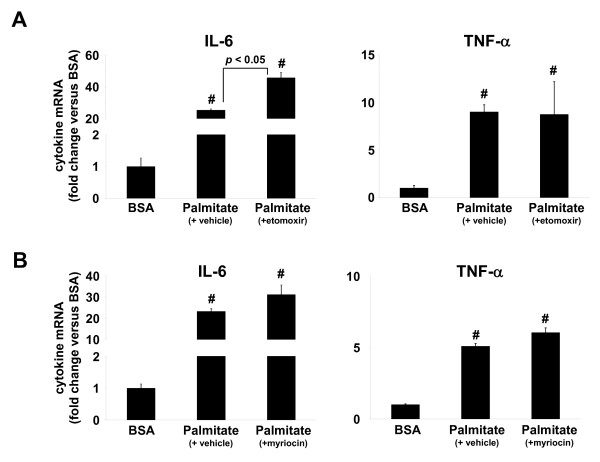
**Mitochondrial oxidation or ceramide generation is not necessary for induction of IL-6 and TNF-α by palmitate**. IL-6 and TNF-α mRNA expression in THP-1 cells treated with *A*, etomoxir (5 μM), an inhibitor of mitochondrial fatty-acyl CoA uptake, or *B*, myriocin, an inhibitor of ceramide biosynthesis, for 1 hour followed by incubation with palmitate (250 μM) for 12 hours. Values are means ± SE (n = 3). #, *p *< 0.01 *versus *BSA.

To determine which intracellular fatty acid metabolic pathways might be involved in the induction of IL-6 and TNF-α by NEFA, THP-1 cells were treated with inhibitors of fatty acid oxidation or ceramide biosynthesis prior to incubation with palmitate and the production of IL-6 and TNF-α mRNAs measured. Etomoxir is an irreversible inhibitor of carnitine palmitoyltransferase I (CPT I), the rate-limiting enzyme for fatty acyl-CoA uptake into mitochondria, where β-oxidation takes place. Pre-treatment of THP-1 cells with 5 μM etomoxir significantly increased IL-6 mRNA production, but not that of TNF-α, in response to palmitate when compared to cells incubated with palmitate in the absence of etomoxir (46-fold IL-6 induction by palmitate + etomoxir versus 25-fold induction with palmitate only, Figure 3A, *p *< 0.05).

Ceramides have been implicated in the induction of inflammatory and cell death signalling pathways in numerous cell types [25]. THP-1 cells were pre-treated with 50 nM myriocin, an inhibitor of serine palmitoyltransferase, the rate-limiting first step in the *de novo *ceramide biosynthetic pathway, prior to incubation with palmitate. IL-6 or TNF- α mRNA induction by palmitate was not affected by myriocin (Figure 3B). Together, these results seem to rule out the β-oxidation and ceramide pathways in monocytes as having a role in the induction of IL-6 and TNF-α by palmitate, thus indirectly implicating components of the glycerolipid biosynthetic pathway in mediating the induction of IL-6 and TNF-α by palmitate.

### Insulin synergizes with palmitate to induce IL-6 mRNA production

**Figure 4:**
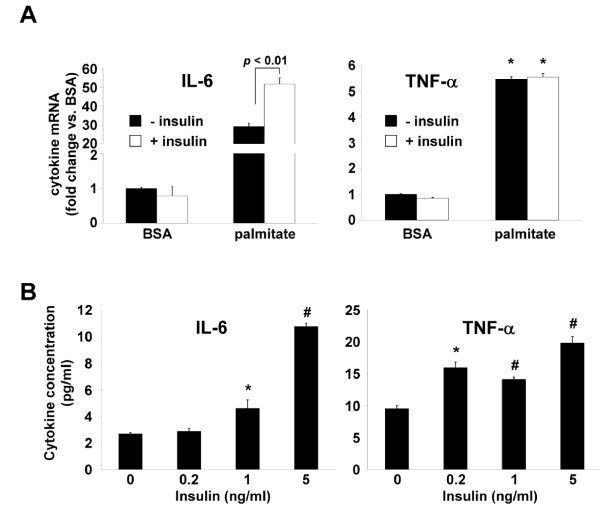
**Synergistic induction of IL-6 by palmitate and insulin**. *A*, IL-6 and TNF-α mRNA expression in THP-1 cells stimulated for 30 minutes with 5 ng/ml insulin (+ insulin) or vehicle (- insulin) and then stimulated with palmitate (250 μM) or fatty acid-free BSA, for an additional 12 hours. *B*, IL-6 and TNF-α concentration in media from THP-1 cells stimulated for 30 minutes with the indicated concentrations of insulin and then stimulated with palmitate (125 μM) for 24 hours. Values are means ± SE (n = 3). *, *p *< 0.05, #, p < 0.01 *versus *(- insulin).

Insulin is an important physiological regulator of intracellular fatty acid metabolism by inhibiting fatty acid oxidation and promoting the synthesis and storage of glycerolipids in adipocytes and other insulin-responsive cells. Therefore, if the glycerolipid biosynthetic pathway is indeed involved in IL-6 production in response to NEFA, insulin may further augment IL-6 production in the presence of excess NEFA. Peripheral blood monocytes would encounter conditions of hyperinsulinemia and above normal concentrations of fatty acids in insulin resistant individuals; therefore, this metabolic situation may contribute the proinflammatory state that is associated with insulin resistance *in vivo*. THP-1 cells were incubated with palmitate ± insulin, or BSA ± insulin, and cellular IL-6 and TNF-α mRNAs measured. In the presence of insulin, palmitate induced significantly more IL-6 mRNA as compared to cells incubated with palmitate alone (52-fold IL-6 induction palmitate + insulin vs. 29-fold IL-6 induction palmitate only, *p *< 0.05, Figure 4A), while insulin had no effect on IL-6 production in cells incubated with BSA. Insulin had no effect on TNF-α production in cells incubated with palmitate or BSA (Figure 4A). IL-6 and TNF-α protein secretion was measured in THP-1 cells incubated with palmitate alone, or palmitate plus varying concentrations of insulin chosen to approximate normal physiologic and hyperinsulinemic conditions. THP-1 cells incubated with palmitate plus insulin at 1 ng/ml and 5 ng/ml concentrations produced significantly more IL-6 protein than cells incubated with palmitate only (Figure 4B), consistent with earlier observations for IL-6 mRNA and demonstrating that physiological concentrations of insulin can synergize with physiological concentrations of palmitate to induce IL-6. In contrast to results obtained with TNF-α mRNA, TNF-α protein secretion from THP-1 cells was higher in cells incubated with insulin and palmitate compared to those incubated with palmitate only (Figure 4B).

**Figure 5:**
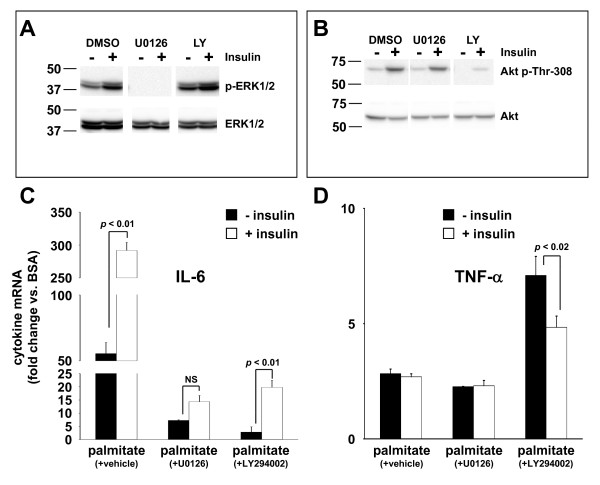
**MEK/ERK and PI3K/Akt signaling pathways regulate IL-6 production**. Total and phosphorylated ERK1/2 (*A*) or total and phosphorylated Akt (*B*) were analyzed by Western blotting of cellular extracts from control (-) and insulin-stimulated (+) THP-1 cells that were pre-treated with vehicle (DMSO), MEK1/2 inhibitor (U0126), or PI3K inhibitor (LY294002). Western blots representative of three independent experiments are shown. IL-6 (*C*) and TNF-α (*D*) mRNA expression determined by qRT-PCR in THP-1 cells treated for 1 hour as in (*A*) followed by stimulation for 30 minutes with 5 ng/ml insulin (+ insulin) or vehicle (- insulin), in the presence of vehicle (DMSO), and then stimulated with palmitate (250 μM) or fatty acid-free BSA, for an additional 24 hours. Values are means ± SE (n = 3) expressed relative to the expression level of these cytokines in BSA-treated cells.

Insulin binding to the insulin receptor engages two primary signal transduction pathways, the mitogen activated protein kinase (MAPK)/extracellular regulated kinase (ERK) kinase (MEK) - ERK pathway and phosphatidylinositide 3-kinase (PI3K) - Akt pathway, in insulin responsive cells [26]. To determine whether insulin signal transduction pathways were activated in THP-1 cells and whether MEK or PI3K inhibitors were effective at inhibiting insulin signal transduction, THP-1 cells were pre-incubated with vehicle (DMSO), a MEK inhibitor (U0126), or a PI3K inhibitor (LY294002) and then were either left untreated or were stimulated for 30 minutes with insulin (5 ng/ml). Cell extracts were analyzed by Western blot using antibodies directed against total ERK1/2 (i.e., phosphorylated and unphosphorylated forms), phosphorylated ERK1/2, total Akt, and phosphorylated Akt. Insulin stimulated phosphorylation of ERK1/2 and Akt was significantly increased over basal levels in the presence of DMSO (Figure 5A, B). U0126, which inhibits MEK1/2, the kinase responsible for phosphorylating ERK1/2, reduced ERK1/2 phosphorylation to undetectable levels in both basal and insulin-stimulated cells, while the PI3K inhibitor LY294002 appeared to slightly increase basal and insulin-stimulated ERK1/2 phosphorylation (Figure 5A). LY294002, an inhibitor of PI3K, the kinase responsible for phosphorylating threonine 308 within Akt, appeared to completely eliminate basal Akt phosphorylation and partially inhibited insulin-stimulated phosphorylation of Akt (Figure 5B).

Inhibition of MEK1/2 or PI3K significantly reduced IL-6 mRNA induction by palmitate ± insulin by approximately 90% (Figure 5C) when compared to DMSO treated cells. However, only the MEK1/2 inhibitor reduced the apparent synergism between palmitate and insulin such that IL-6 production in cells treated with palmitate + insulin was not significantly greater than in cells treated only with palmitate (Figure 5C).

Inhibition of MEK1/2 had little effect on TNF- α production in cells treated with palmitate ± insulin, however, PI3K inhibition with LY294002 significantly increased TNF-α production in palmitate-treated cells versus cells treated with palmitate + vehicle (DMSO) (Figure 5D). Interestingly, this effect of LY294002 was partially reversed by insulin co-treatment, perhaps due to the incomplete inhibition of Akt phosphorylation that was achieved in these cells as demonstrated in Figure 5B.

### Insulin synergizes with palmitate to induce IL-6 mRNA in primary human monocytes

**Figure 6:**
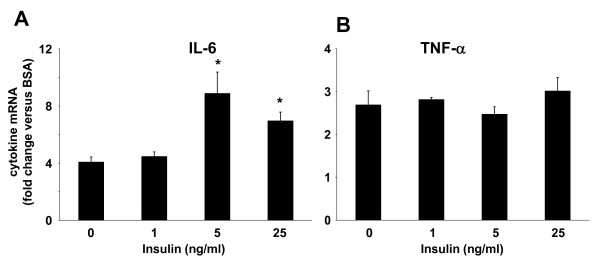
**Synergistic induction of IL-6 mRNA in primary human monocytes**. Monocytes isolated by adherence from human PBMC fraction of whole blood were incubated for 30 minutes with varying concentrations of insulin, followed by 24 hour incubation with 500 μM palmitate or BSA. IL-6 (A) and TNF-α (B) mRNAs were determined by qRT-PCR. Values are means ± SE (n = 3) expressed relative to the expression level of these cytokines in BSA-treated cells. *, *p *< 0.05 *versus *(- insulin).

Although THP-1 cells have been used extensively as a model for primary human monocytes, in some instances the responses exhibited by THP-1 cells and primary cells do not correspond. To determine whether primary human monocytes produce IL-6 and TNF-α in response to incubation with saturated NEFA, and whether saturated NEFA and insulin synergize to induce IL-6 in these cells, primary monocytes isolated from the PBMC fraction of whole blood were exposed to palmitate ± insulin for 24 hours and the production of IL-6 and TNF-α mRNA determined by qRT-PCR. Similar to our observations in THP-1 cells, albeit with a lower magnitude, palmitate stimulated IL-6 and TNF-α mRNA expression (Figure 6A, B). Furthermore, insulin, when used at concentrations similar to those used in THP-1 cells, augmented the production of IL-6 in response to palmitate to a similar degree as in THP-1 cells (Figure 6A), while TNF-α mRNA levels were not affected by the presence of insulin (Figure 6B).

## Discussion

These studies are the first to demonstrate that human monocytes synthesize and secrete IL-6 and TNF-α in response to saturated NEFA. Our results are consistent with the observations of others that saturated NEFA can induce an inflammatory response in a variety of other cell types, including endothelial cells [27], adipocytes [28], myotubes [29], and macrophages [30,31]. NEFA have recently been shown to increase reactive oxygen species and the expression of β2 integrin in monocytes, and increase monocyte adhesion to endothelial cells [32]. Our results suggest that circulating blood monocytes *in vivo *may respond to increases in saturated NEFA concentrations in insulin resistant conditions by producing high levels of IL-6, which could prime these cells to generate a robust local or systemic inflammatory response and contribute to the development of complications such as T2DM and atherosclerosis.

Regarding the molecular mechanism of inflammatory cytokine induction by saturated NEFA, some studies have demonstrated that saturated NEFA induce IL-6 via TLR2 or TLR4 receptors in myocytes, macrophages, and adipocytes [19,33]. However, other studies have demonstrated that NEFA metabolism was required for the induction of inflammatory cytokines in endothelial cells [27]. Therefore, it appears that saturated NEFA are potent inducers of inflammation in diverse cell types, but that the molecular mechanisms for cytokine induction vary according to cell type. Our results suggest that monocytes are more like endothelial cells in their inflammatory response to saturated NEFA, in that fatty acid metabolism appears to be required for cytokine induction (Figure 2B). In fact, our results with the β-oxidation inhibitor etomoxir showed that inhibition of β-oxidation of palmitate enhanced IL-6 induction in monocytes. This raises the interesting possibility that interference with β-oxidation may increase the intracellular concentration of palmitoyl-CoA available for use by other metabolic pathways that can stimulate IL-6 mRNA production. A candidate for this IL-6 inducing pathway is the triglyceride synthesis pathway, which contains several intermediates such as lysophosphatidic acid, phosphatidic acid, and diacylglycerol, which have all been shown to have inflammation-promoting properties in a variety of cells [34,35]. Consistent with our studies, Staiger *et al*. [27] showed that neither mitochondrial β-oxidation of fatty acids or ceramide biosynthesis was involved in IL-6 induction by palmitate in endothelial cells. However, Schwartz *et al*. [36] recently reported that palmitate metabolism to ceramide was necessary for amplification of LPS-induced inflammation in human monocytes. Direct measurement of glycerolipid intermediates in monocytes incubated with NEFA will be required to definitively support the hypothesis that increased fatty acid flux through the triglyceride synthesis pathway is involved in the induction of IL-6, TNF- α, and perhaps other cytokines whose levels increase in insulin resistant conditions.

Our results demonstrated that hyperinsulinemia, coupled with elevated levels of NEFA, generated higher levels of IL-6 production in monocytes compared to the IL-6 response to NEFA alone. The marked increase in IL-6 production observed between 0, 1 and 5 ng/ml concentrations of insulin demonstrates that even modest increases in insulin levels within the range encompassing fasting and post-prandial insulin levels observed in insulin resistant subjects, along with physiological levels of NEFA, can dramatically affect IL-6 production. This result is particularly relevant in the context of the metabolic status of the obese, insulin resistant individual, where fasting levels of NEFA and insulin are likely to be chronically elevated [1]. Numerous studies have shown that IL-6 concentration is increased in the circulation of obese, insulin resistant humans [37]. Kern *et al*. [9] implicated NEFA and insulin in the regulation of circulating IL-6 by demonstrating that circulating IL-6 levels showed a strong positive correlation with serum NEFA levels and a strong negative correlation with insulin sensitivity, which varies inversely with insulin levels.

Although IL-6 plays a deleterious role in the development of coronary artery disease and is an early indicator of incipient type 2 diabetes mellitus [38-40], some studies suggest that IL-6 may have beneficial effects in the resolution of inflammation and improvement of insulin sensitivity following exercise [39,41,42]. Some positive effects of IL-6 have been postulated to occur via activation of adenosine monophosphate-activated protein kinase (AMPK) [43], an enzyme whose activation promotes ATP production via fatty acid oxidation. Thus, increased IL-6 production from exercised muscle may serve as a local signal to increase energy production via fatty acid oxidation, whereas increased IL-6 production from peripheral monocytes exposed to elevated concentrations of fatty acids may serve as a systemic signal to inhibit fatty acid production and stimulate fatty acid oxidation. Indeed, a recent study demonstrated that mice lacking IL-6 were prone to develop hepatosteatosis, liver inflammation and insulin resistance when compared to wild-type mice [44], supporting a role for IL-6 in the suppression of inflammation and regulation of lipid homeostasis and metabolism *in vivo*.

Insulin signaling in the monocyte/macrophage lineage may play a vital role in the regulation of local and systemic inflammation. In mice, selective removal of insulin receptor expression in the myeloid lineage, which includes monocytes/macrophages and granulocytes, abolished LPS-elicited IL-6 production in macrophages, while minimally affecting TNF-α or MCP-1 production [45]. A recent report demonstrated that mice lacking insulin receptor expression in the myeloid lineage were protected from insulin resistance when fed a high-fat diet, possibly due to reduced systemic inflammation and decreased monocyte/macrophage infiltration of white adipose tissue [46]. Evidence from *in vitro *studies also supports a role for insulin in the regulation of proinflammatory cytokine production in macrophages. LPS treatment of monocytes generated a greater IL-6 response when co-administered with insulin compared to the IL-6 response to LPS treatment alone [47]. Therefore, our results and the results of others support the hypothesis that IL-6 induction by NEFA in monocytes and macrophages is regulated by insulin.

It may seem counterintuitive to postulate that some of the cellular effects of insulin could be preserved in the face of insulin resistance. However, insulin resistance specifically refers to the inability of insulin to promote cellular glucose uptake, which is mediated primarily via the phosphatidylinositol-3 kinase (PI3K) signalling pathway downstream of the insulin receptor. It has been documented that the PI3K pathway appears to be selectively inhibited in insulin resistant states, whereas another major signalling pathway downstream of the insulin receptor, the Ras-Raf-MAPK signaling pathway, remains sensitive to insulin even when metabolic effects of insulin are blunted in some cell types [48,49]. Our results demonstrate that both PI3K-Akt and MAPK signalling pathways are utilized by palmitate to produce IL-6 and TNF-α. However, the MAPK signalling pathway seems to be more important for the synergistic induction of IL-6 by palmitate and insulin.

Some limitations of this study warrant discussion. THP-1 cells used in this study are transformed cells that were derived from an acute myelogenous leukemia patient. Although the primary findings obtained in THP-1 cells were validated in primary human monocytes *ex vivo*, further studies are necessary to determine whether our findings *in vitro *can be translated to *in vivo *conditions. Insulin resistance results in numerous metabolic abnormalities in addition to increased NEFA concentrations and hyperinsulinemia that were modelled in isolation in this study, therefore further *in vitro *studies will be required to understand how the complex metabolic alterations of insulin resistance regulate inflammation in human monocytes.

## Conclusions

The proinflammatory subset of monocytes has the capacity to produce large quantities of inflammatory cytokines such as IL-6, and this subset of monocytes accumulates in adipose tissue and artery walls, where they are believed to initiate and propagate disease processes. Thus, chronic activation of monocyte IL-6 production by high levels of fatty acids and hyperinsulinemia in insulin resistant subjects could produce local and systemic inflammation. Local production of IL-6 at sites of monocyte infiltration could initiate insulin resistance in adipose tissue, or produce rupture-prone atherosclerotic plaques in arteries. Systemic production of IL-6 could induce the acute phase response in liver, which entails the production of pro-coagulant factors such as plasminogen activator inhibitor 1 and antimicrobials such as C-reactive protein, whose increased concentrations have been associated with cardiovascular disease [50,51]. Alternatively, monocyte production of IL-6 may be beneficial, serving to suppress inflammatory stress induced by NEFA and other metabolites. A better understanding of the molecular mechanisms utilized by saturated NEFA and insulin to regulate IL-6 production in proinflammatory monocytes could identify targets for novel anti-inflammatory molecules that could reduce the incidence of complications from insulin resistance.

## Competing interests

The authors declare that they have no competing interests.

## Authors' contributions

RCB conceived and designed the studies, performed experiments, collected and analyzed data, and drafted the manuscript. GEC performed Luminex bioassays for IL-6 and TNF-α. YO contributed to data collection and analysis. KMT contributed to data analysis. CKL contributed to drafting the manuscript. JLF contributed to the design of the studies, analysis of data, and drafting the manuscript. All authors read and approved the final submitted version of the manuscript.
